# Selective Confinement by a COF‐Derived Sub‐Nanoporous Interface for High‐Performance CoF_2_ Thermal Battery Cathodes

**DOI:** 10.1002/advs.202521241

**Published:** 2026-01-04

**Authors:** Mengfan Xu, Jun Zhang, Lili Zhao, Ying Chu, Furui Luo, Xinping Cao, Shengnan Guo, Xueying Wang, Yongping Zhu, Song Wang

**Affiliations:** ^1^ University of Chinese Academy of Sciences Beijing P. R. China; ^2^ State Key Laboratory of Mesoscience and Engineering Institute of Process Engineering Chinese Academy of Sciences Beijing P. R. China

**Keywords:** covalent organic frameworks, selective confinement, sub‐nanopore, thermal battery, transition metal fluoride cathodes

## Abstract

The pervasive dissolution of transition metal fluoride (TMF) cathodes presents a fundamental barrier to their application in high‐voltage thermal batteries and other Li^+^‐conducting systems. Herein, we report a novel selective confinement strategy inspired by ion sieving to overcome this challenge by constructing a sub‐nanoporous carbon interface in situ on CoF_2_ particles. Derived from a covalent organic framework (COF), this interface features precisely defined 0.54 nm pores that exploit the size difference between Li^+^ ions (∼0.15 nm) and dissolved transition metal fluoride derived complex ions (∼0.8 nm), effectively confining the active material while enabling unimpeded ionic conduction. This tailored design successfully suppresses cathode shuttling effect, enabling a thermal battery that delivers an exceptional discharge plateau >2.5 V, a high specific capacity of 365 mAh g^−1^, and a remarkable specific energy of 882 Wh kg^−1^ at 100 mA cm^−2^. Mechanism studies confirm the dissolved transition metal fluoride derived complex ions as CoCl_4_^2−^ and efficient confinement of it. This work provides a general and effective interface engineering strategy for unlocking the full potential of metal fluoride cathodes in advanced energy storage.

## Graphical abstract

Dissolution and shuttling of TMF cathodes hinders high‐voltage thermal batteries. A novel selective confinement strategy using a sub‐nanoporous COF‐derived carbon interface (0.54 nm pores) on CoF_2_ suppresses cathode material shuttling. Size‐selective confinement blocks large dissolved ions (∼0.8 nm) while allowing Li⁺ passage, enhancing thermal battery performance with high‐voltage, capacity, and energy density.

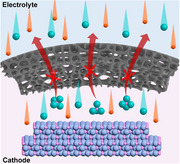

## 1 Introduction

A thermal battery is a type of thermally activated primary battery, primarily used in military applications such as power sources for weapon systems [1, 2, 3]. Leveraging molten salt electrolytes, thermal batteries offer ultrahigh power output, excellent safety performance, and an ultralong shelf life. However, with the continuous advancement of modern weapon systems, conventional metal sulfide‐based systems are increasingly unable to meet the demand for higher voltage and power output. For instance, traditional sulfides like FeS_2_, CoS_2_, and NiS_2_ exhibit discharge plateaus below 2 V [4, 5, 6, 7, 8]. More critically, these sulfides are prone to thermal decomposition at operating temperatures exceeding 550°C, leading to reduced discharge capacity and increased safety risks.

Recently, transition metal fluorides (TMFs), which operate via conversion reactions, have garnered significant attention due to their high theoretical operating voltages—a result of the strong electronegativity of fluoride ions coupled with exceptional thermal stability [9, 10]. Considered promising next‐generation cathode materials, TMFs offer high gravimetric capacities (e.g., 712 mAh g^−1^ for FeF_3_, 553 mAh g^−1^ for CoF_2_, and 528 mAh g^−1^ for CuF_2_), high theoretical voltages (2.74 V for FeF_3_, 2.85 V for CoF_2_, and 3.55 V for CuF_2_), and the ability to operate stably at temperatures up to 800°C [11, 12]. Nonetheless, TMFs suffer from limitations such as poor electronic conductivity, complex and costly synthesis routes, and dissolution in electrolytes, which hinder their practical application not only in thermal batteries but also in other lithium‐ion conducting systems like lithium‐ion batteries (LIBs) [13, 14]. Dissolution is particularly problematic in thermal batteries due to the high operating temperatures and strong solvation power of molten salts, causing significant degradation in discharge performance and failure to reach theoretical potentials [15]. To date, limited progress has been made in addressing these challenges in thermal battery systems, primarily owing to their inherent complexities.

Several strategies have been proposed to mitigate TMF dissolution and shuttling in LIBs. Most methods focus on constructing a cathode‐electrolyte interphase (CEI) between the TMF cathode and the electrolyte [16, 17]. For example, a dense NiO layer has been applied to CuF_2_ electrodes to prevent direct contact with the electrolyte [18]. However, such dense CEI layers increase lithium‐ion transport resistance, resulting in sluggish reaction kinetics unsuitable for the ultrahigh power output required in thermal batteries. Alternatively, nanoporous carbon interlayers have been used to encapsulate TMFs like CoF_2_ and FeF_3_ for high‐rate LIBs [19, 20]. Nevertheless, the large pore sizes in these matrices are ineffective at preventing TMF dissolution in thermal batteries, given the small ion size and extremely low viscosity of molten salt electrolytes [21]. Recently, hydrogel layers have also been explored as barriers between TMFs and organic electrolytes, utilizing strong chelation interactions between metal ions and sodium alginate to confine active materials [22, 23]. Unfortunately, these hydrogels cannot withstand the high operating temperatures characteristic of thermal batteries. Therefore, it is crucial to develop new strategies specifically tailored to the unique features of thermal batteries to address the dissolution and shuttling of active materials, thereby enabling the development of next‐generation high‐voltage, high‐power‐density thermal batteries.

**FIGURE 1:**
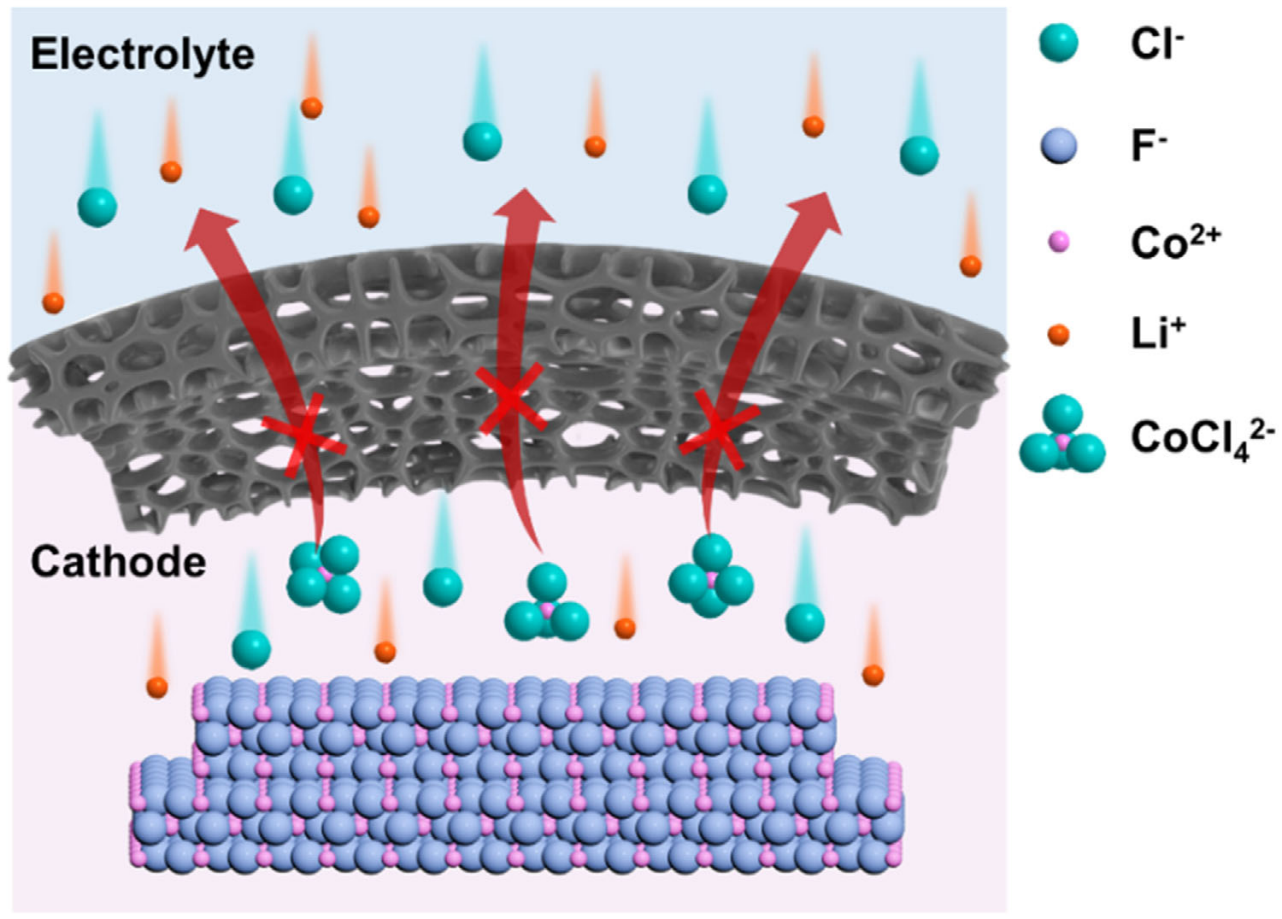
Illustration of size selective transmission of ions between electrolyte and cathode enabled by sub‐nanoporous interface.

In this work, we introduce the concept of an ion sieve [24] in the construction of the cathode‐electrolyte interphase by exploiting the significant size difference between dissolved metal fluoride‐based complex ions (e.g., CoCl_4_^2−^, ∼0.8 nm) [25, 26] and charge‐carrying lithium ions (0.15 nm) [27] in molten salt electrolytes (Figure 1). A covalent organic framework (COF)‐derived sub‐nanoporous interface with a uniform sub‐nano pore size of 0.54 nm was constructed in situ on the surface of a fluoride cathode. This design effectively confines metal fluoride‐derived complex ions within the cathode region while allowing unimpeded transport of conductive ions. Consequently, by effectively addressing the dissolution and shuttling of TMFs in molten salt electrolytes, we achieved a high discharge voltage plateau exceeding 2.5 V and a high specific capacity of over 360 mAh g^−1^ (cutoff voltage of 2 V) at a current density of 100 mA cm^−^^2^ for a thermal battery using CoF_2_ as the cathode material.

## 2 Results and Discussion

### 2.1 COF‐Derived Sub‐Nanoporous Carbon Materials

Porous materials with narrow pore size distributions are demanded for the construction of a sub‐nanoporous interface. COFs are typically covalently linked nanoporous materials with precise pore sizes mostly larger than 1 nm [28, 29, 30], which are too large to restrict the mobility of cobalt complex ions [24, 31]. Furthermore, the working temperature of thermal batteries is typically above 500°C, which is near or above the decomposition temperature of most COFs [32, 33, 34]. Thus, we reduced their pore size to the sub‐nanometer scale and simultaneously improved their high‐temperature stability through carbonization [35, 36, 37], obtaining COF‐derived sub‐nanoporous carbon (CSC). TAPB‐BTCA COF was selected due to its small intrinsic pore size and well‐controlled polymerization kinetics suitable for nanocomposite construction. TAPB‐BTCA COF was synthesized via the polymerization of 1,3,5‐tris(4‐aminophenyl)benzene (TAPB) and 1,3,5‐benzenetricarbaldehyde (BTCA) monomers using a Reversible Polycondensation‐Termination (RPT) strategy, following our previous work [38, 39, 40, 41, 42] (Figure S1).

**FIGURE 2:**
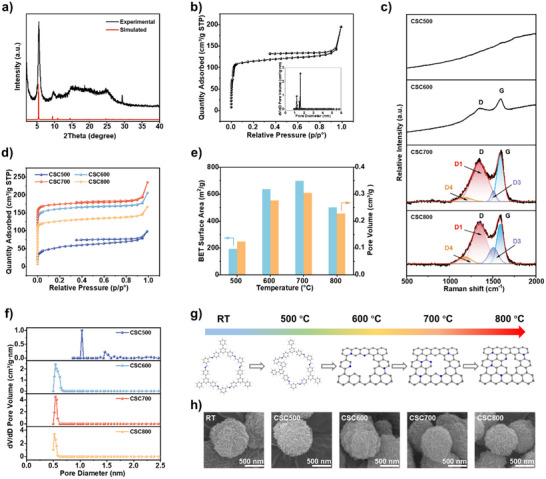
Characterizations of COF‐derived sub‐nanoporous carbons. (a) Experimental and simulated PXRD patterns of TAPB‐BTCA COF. (b) Nitrogen adsorption and desorption isotherms at 77 K and pore width distribution for TAPB‐BTCA COF. (c) Raman spectrums of CSC500, CSC600, CSC700 and CSC800. (d–f) Nitrogen adsorption and desorption isotherms at 77 K (d), BET surface areas and pore volumes (e), and pore width distributions (f) for CSC500, CSC600, CSC700, and CSC800. (g) Structure illustrations for carbonization process of TAPB‐BTCA COF. (h) FESEM images of TAPB‐BTCA COF, CSC500, CSC600, CSC700, and CSC800.

The chemical structure and crystallinity of the as‐synthesized TAPB‐BTCA COF were characterized prior to carbonization. Fourier transform infrared (FT‐IR) spectroscopy confirmed the formation of imine bonds, evidenced by a peak at 1624 cm^−1^ (Figure S2). High crystallinity was demonstrated by powder X‐ray diffraction (PXRD). A strong peak at 5.5°, along with well‐defined high ordered diffraction peaks at 9.45°, 10.9°, and 14.45°, were observed in the PXRD pattern, matching well with the (100), (110), (200), and (210) crystal planes of a simulated A‐A stacking model (Figure 2a) [38]. TAPB‐BTCA COF exhibited a type‐I N_2_ adsorption‐desorption isotherm (Figure 2b). The Barrett‐Emmett‐Teller (BET) surface area was calculated to be 468 m^2^ g^−1^, with a pore volume of 0.23 cm^3^ g^−1^ and a mean pore size of 1.6 nm determined by the nonlocal density functional theory method (Figure 2b). This pore size agrees with the 1.6 nm interplanar spacing calculated from the main XRD peak at 5.5°. Field emission scanning electron microscopy (FESEM) revealed that TAPB‐BTCA COF possesses a pompon‐like spherical morphology composed of interwoven nanoflakes (Figure S3).

COF‐derived sub‐nanoporous carbons were prepared by direct carbonization of the precursor TAPB‐BTCA COF under an argon atmosphere. Thermogravimetric analysis (TGA) showed that TAPB‐BTCA COF undergoes a rapid weight loss centered at 524°C in an inert atmosphere, followed by two moderated weight loss zones around 590°C and 740°C, yielding a stable carbon residue of ∼62% after 800°C (Figure S4). Based on this, carbonization temperatures of 500°C, 600°C, 700°C, and 800°C were selected for CSC fabrication. Four CSCs were fabricated from the TAPB‐BTCA COF precursor at these temperatures, designated as CSC500, CSC600, CSC700, and CSC800, respectively. The graphitization process was monitored by Raman spectroscopy (Figure 2c). Obvious baseline drift due to fluorescence was observed in the spectra of CSC500 and CSC600, indicating the retention of aromatic conjugated structures from the original COF (Figure S5). This fluorescence disappeared in CSC700 and CSC800, suggesting the loss of the aromatic structure. Graphitic structure formation began at 600°C, as confirmed by the appearance of distinct D and G bands around 1350and 1590 cm^−1^, respectively. Peak analysis of CSC700 presented a narrower D1 peak and weaker D4 and D3 peaks, indicating less amorphous and disordered graphitic lattice [43].

The evolution of sub‐nanopores from the original nanopores was studied using N_2_ physisorption. All CSCs exhibited type‐I sorption isotherms with steep nitrogen uptake at low relative pressure (P/P_0_< 0.01), indicative of abundant micropores (Figure 2d). The BET surface area and pore volume initially decreased to 192.6 m^2^ g^−1^ and 0.12 cm^3^ g^−1^ for CSC500 from the original 468 m^2^ g^−1^ and 0.23 cm^3^ g^−1^ of the COF, due to structural degradation at 500°C (Figure 2e). The pore size distribution for CSC500 showed peaks at 1.0 nm and 1.5 nm (Figure 2f), attributed to newly collapsed and residual COF pores, respectively. The BET surface area and pore volume increased with graphitization from 500°C to 700°C, reaching 699 m^2^ g^−1^ and 0.3 cm^3^ g^−1^ for CSC700, even exceeding the values for the precursor COF. This suggests the generation of abundant new sub‐nanopores, evidenced by a narrow pore size distribution centered at 0.54 nm for CSC600 and CSC700. However, further increasing the carbonization temperature to 800°C decreased the BET surface area and pore volume to 502 m^2^ g^−1^ and 0.23 cm^3^ g^−1^, likely due to C─N bond decomposition and pore collapse. The sub‐nanopore evolution is illustrated in Figure 2g: new graphitized structures with sub‐nanopores generated below 600°C, are enhanced at 700°C, and partially degrade at 800°C, consistent with the three weight loss peaks in the TGA curve (Figure S4). Notably, these structural changes occurred primarily at the micro‐scale, as the overall pompon‐like morphology was well preserved during carbonization (Figure 2h; Figure S6), likely because gases generated during carbonization could easily escape through the open pore structure of the COF. This ability to generate sub‐nanopores while maintaining morphology enables the in situ construction of a sub‐nanoporous interface on fluoride cathode surfaces.

### 2.2 Construction of CoF_2_@CSCs

**FIGURE 3:**
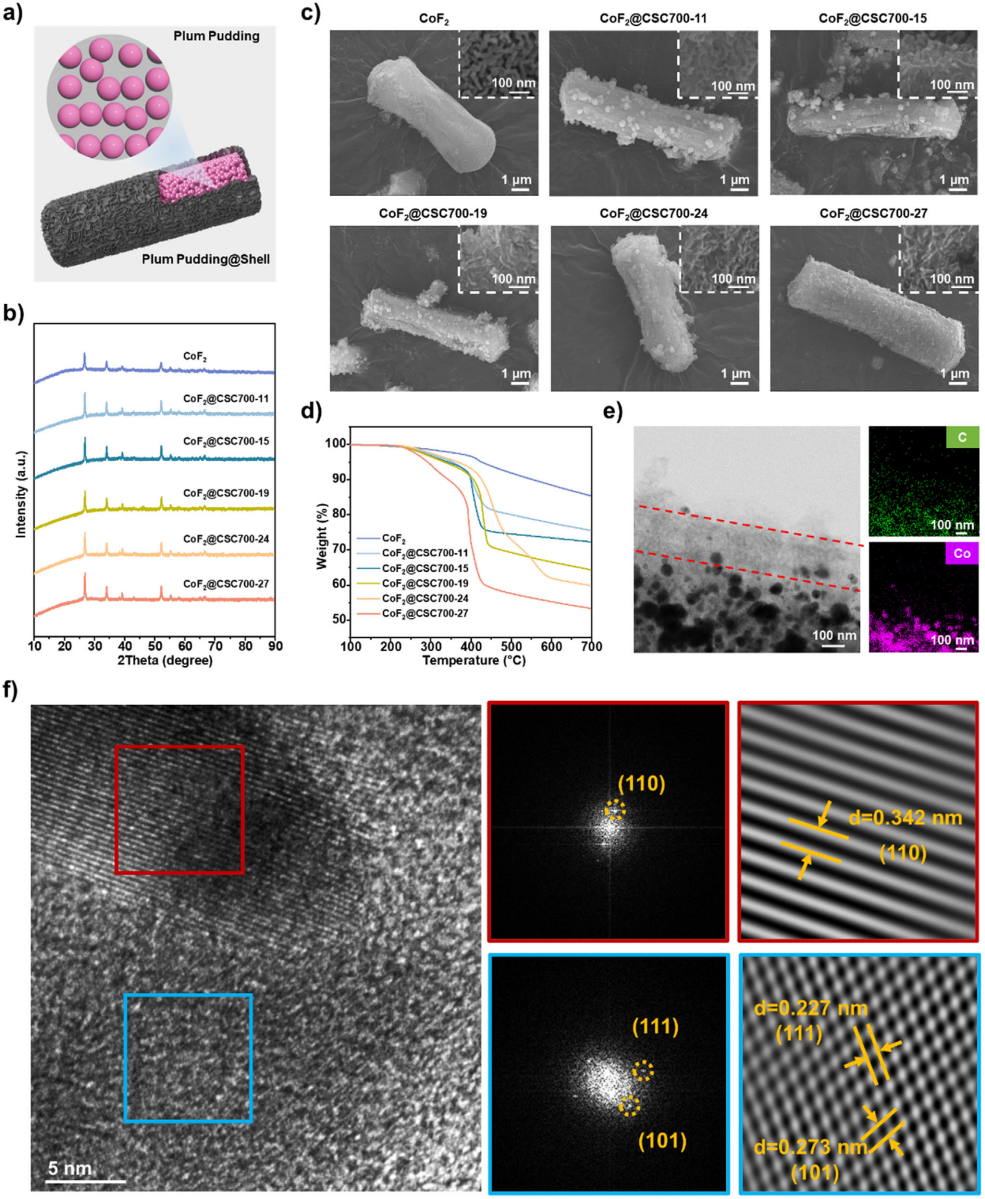
Characterizations of CoF_2_@CSC700s. (a) Illustration of plum pudding@shell structure of the CoF_2_@CSC700 composites. (b–d) XPD patterns (b), FESEM images (c), and TGA thermograms (d) of bared CoF_2_ and CoF_2_@CSC700s. (e) TEM and EDS elemental mapping images of CoF_2_@CSC700‐24. (f) HRTEM, FFT, and reversed FFT images for CoF_2_@CSC700‐24.

To better confine dissolved transition metal fluoride derived complex ions within the cathode region, a plum pudding@shell structure (Figure 3a) was designed to thoroughly cover cathode materials, ensuring confinement under harsh discharge conditions of high‐temperature and current density. CoF_2_ nanoparticles were selected as the fluoride cathode due to their high theoretical voltage and specific capacity [11, 44, 45]. The CoF_2_ nanoparticles were first synthesized via fluorosilicate decomposition at 260°C (Figures S7–S9). High crystallinity was confirmed by sharp XRD diffraction peaks (Figure 3b). The CoF_2_ particles exhibited a micro‐sized rod‐like assembly structure composed of nanorods with a diameter of 20 nm and a length of 80 nm (Figure 3c; Figure S10). After dispersing the CoF_2_ particles into the TAPB‐BTCA COF polymerization solution and carefully controlling the polymerization kinetics via the RPT strategy, the COF formed not only an outer shell around the micro‐assemblies but also filled the gaps between the CoF_2_ nanorods (Figure S11). The final CoF_2_@CSC plum pudding@shell structures were obtained after carbonization in argon and subsequent fluorination with NF_3_. The CSC content was tuned by varying the concentration of CoF_2_ particles in the COF polymerization solution and confirmed by elemental analysis. Five CoF_2_@COF composites with different CSC contents (11, 15, 19, 24, and 27 wt.%) were prepared after carbonization at 700°C, designated as CoF_2_@CSC700‐11, CoF_2_@CSC700‐15, CoF_2_@CSC700‐19, CoF_2_@CSC700‐24, and CoF_2_@CSC700‐27, respectively. CoF_2_ component were confirmed as high crystalline by XRD patterns with sharp characteristic peaks (Figure 3b; Figure S12). The different CSC contents were also evidenced by TGA in air, showing a sharp weight loss after approximately 400°C (Figure 3d). XRD confirmed that all CoF_2_@CSC700 composites contained highly crystalline CoF_2_, with diffraction peaks matching its standard PDF card (PDF#76‐0652) (Figure 3b; Figure S12).

The plum pudding@shell structure of the CoF_2_@CSC700 composites was confirmed by electron microscopies. At low CSC content, the CSC primarily filled the gaps between the CoF_2_ nanorods (Figure 3c; Figure S13). As the CSC content increased, a continuous shell layer with the characteristic nanoflake morphology of TAPB‐BTCA COF became increasingly evident on the exterior of the micro‐rod assemblies. For representative CoF_2_@CSC700‐24, TEM imaging and energy‐dispersive X‐ray spectroscopy (EDS) elemental mapping (Figure 3e) showed good overlap of Cobalt (Co) and Carbon (C) signals within the CoF_2_ assembly zone, consistent with the plum pudding structure. A shell region of ∼200 nm thickness, consisting primarily of carbon, was observed surrounding the assembly. High‐resolution transmission electron microscopy (HRTEM) images of CoF_2_@CSC700‐24 further confirmed the structure, showing crystalline CoF_2_ nanoparticles surrounded by amorphous carbon. Selected area fast Fourier transform (FFT) and inverse FFT patterns revealed lattice fringes with spacings of 0.342, 0.227, and 0.273 nm, corresponding to the (110), (111), and (101) crystal planes of CoF_2_ (PDF#76‐0652), respectively.

### 2.3 Electrochemical Performance of the CoF_2_@CSCs Cathodes

**FIGURE 4:**
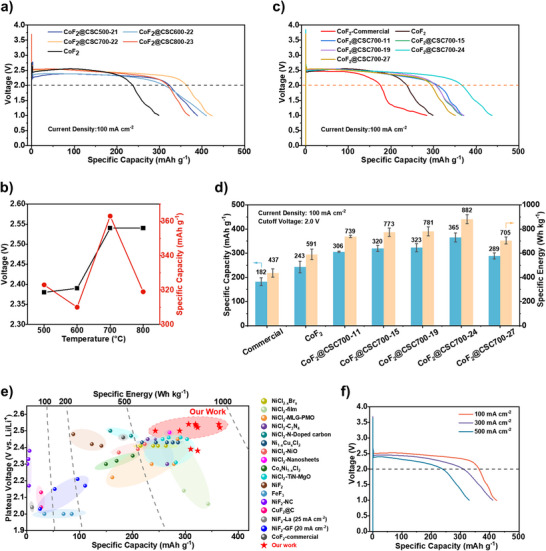
Electrochemical performances of CoF_2_@CSC composites as cathodes. (a,b) Discharge profiles of voltage vs. specific capacity (based on CoF_2_ active mass) (a) and their summary (b) for bared CoF_2_, CoF_2_@CSC500‐21, CoF_2_@CSC600‐22, CoF_2_@CSC700‐22, and CoF_2_@CSC800‐23 cathodes. The discharge current density was kept constant at 100 mA cm^−2^. Specific capacity was based on 2.0 V cut off voltage. (c,d) Discharge profiles of voltage vs. specific capacity (based on CoF_2_ active mass) (c) and their summary (d) for commercial CoF_2_, bared CoF_2_, CoF_2_@CSC700‐11, CoF_2_@CSC700‐15, CoF_2_@CSC700‐19, CoF_2_@CSC700‐24, and CoF_2_@CSC700‐27. Specific capacity and energy are both based on 2.0 V cut off voltage. (e) Ashby chart comparing plateau voltages, specific capacities, and specific energies with reported data in the literature. Data is according to the literatures: NiCl_2‐x_Br_x_ [46], NiCl_2_‐film [47], NiCl_2_‐MLG‐PMO [48], NiCl_2_‐C_3_N_4_ [49], NiCl_2_‐N‐Doped Carbon [50], Ni_1‐x_Cu_x_Cl_2_ [1], NiCl_2_‐NiO [51], NiCl_2_‐Nanosheets [52], Co_x_Ni_1‐x_Cl_2_ [53], NiCl_2_‐TiN‐MgO [54], NiF_2_ [55], FeF_3_ [56], NiF_2_‐NC [57], CuF_2_@C [58], NiF_2_‐La [59], NiF_2_‐GF [60], and CoF_2_‐commercial. (f) Discharge profiles of voltage vs. specific capacity at different constant current densities for CoF_2_@CSC700‐24 cathode.

The electrochemical performances of CoF_2_@CSC composites as cathodes were evaluated in single‐cell thermal batteries using LiCl‐LiF‐Li_2_SO_4_ electrolyte and a Li‐B alloy anode. Discharge profiles (voltage vs. specific capacity based on CoF_2_ active mass) were recorded at a constant current density of 100 mA cm^−2^ and a temperature of 500°C. First, composites with similar CSC content (∼21–23 wt.%) but carbonized at different temperatures (CoF_2_@CSC500‐21, CoF_2_@CSC600‐22, CoF_2_@CSC700‐22, CoF_2_@CSC800‐23) were tested to evaluate the influence of the carbon structure (Figure S14). All discharge curves showed a single dominant voltage plateau followed by a sharp drop to 1.0 V. The voltage plateaus for CoF_2_@CSC500‐21 and CoF_2_@CSC600‐22 were 2.38 and 2.39 V, respectively, lower than the 2.46 V observed for an unmodified CoF_2_ cathode (Figure 4a,b), which we attribute to the poor electronic conductivity of the amorphous carbon formed at lower carbonization temperatures. This issue was significantly alleviated in composites carbonized at 700°C and 800°C, where graphitization dominated, resulting in higher discharge plateaus of 2.54 V for both CoF_2_@CSC700‐22 and CoF_2_@CSC800‐23, exceeding that of bare CoF_2_. Furthermore, all CoF_2_@CSC cathodes delivered higher specific capacities (measured at a 2.0 V cutoff) than the bare CoF_2_ cathode (243 mAh g^−1^), which we attribute to the confinement effect of the CSC layer (discussed later). Among them, CoF_2_@CSC700‐22 exhibited the best performance, delivering a specific capacity of 363 mAh g^−1^ at the 2.0 V cutoff (Figure 4a,b), likely due to the largest pore volume and specific surface area of CSC700. Based on these results, 700°C was selected as the optimal carbonization temperature.

A thicker CSC interface should better confine complex ions but may also increase Li^+^ diffusion resistance. To optimize this trade‐off, the electrochemical performance of CoF_2_@CSC700 composites with different CSC contents (11–27 wt.%) was evaluated. Their discharge curves are shown in Figure 4c (see three replicates for each curve in Figure S15) and summarized in Figure 4d. All CoF_2_@CSC700 composites showed higher voltage plateaus (2.50–2.54 V) compared to bare CoF_2_, indicating enhanced conductivity from CSC‐700. All composites also exhibited higher specific capacities (at 2.0 V cutoff) than both synthesized and commercial CoF_2_, with a maximum of 365 mAh g^−1^ for CoF_2_@CSC700‐24. The specific energy also peaked for CoF_2_@CSC700‐24 at 882 Wh kg^−1^, which is 49.2% and 101.8% higher than the synthesized and commercial CoF_2_ references, respectively. An Ashby chart comparing the discharge performance of this work against other high‐voltage thermal battery cathode materials from the literature is presented in Figure 4e. To the best of our knowledge, our CoF_2_@CSC700 cathodes demonstrate superior electrochemical performance, with CoF_2_@CSC700‐22 and CoF_2_@CSC700‐24 showing the highest specific capacity and specific energy with plateaus above 2.5 V, highlighting their potential as cathode systems for next‐generation thermal batteries.

The discharge performance of CoF_2_@CSC700‐24 under high current densities is shown in Figure 4f. The voltage plateaus decreased slightly to 2.45 V and 2.39 V at 300 and 500 mA cm^−2^, respectively, due to increased concentration polarization. The specific capacities decreased to 308 and 240 mAh g^−1^ at these current densities, demonstrating considerable tolerance for high‐rate discharge.

### 2.4 CoF_2_ Dissolution Mechanism and Suppression by CSC

**FIGURE 5:**
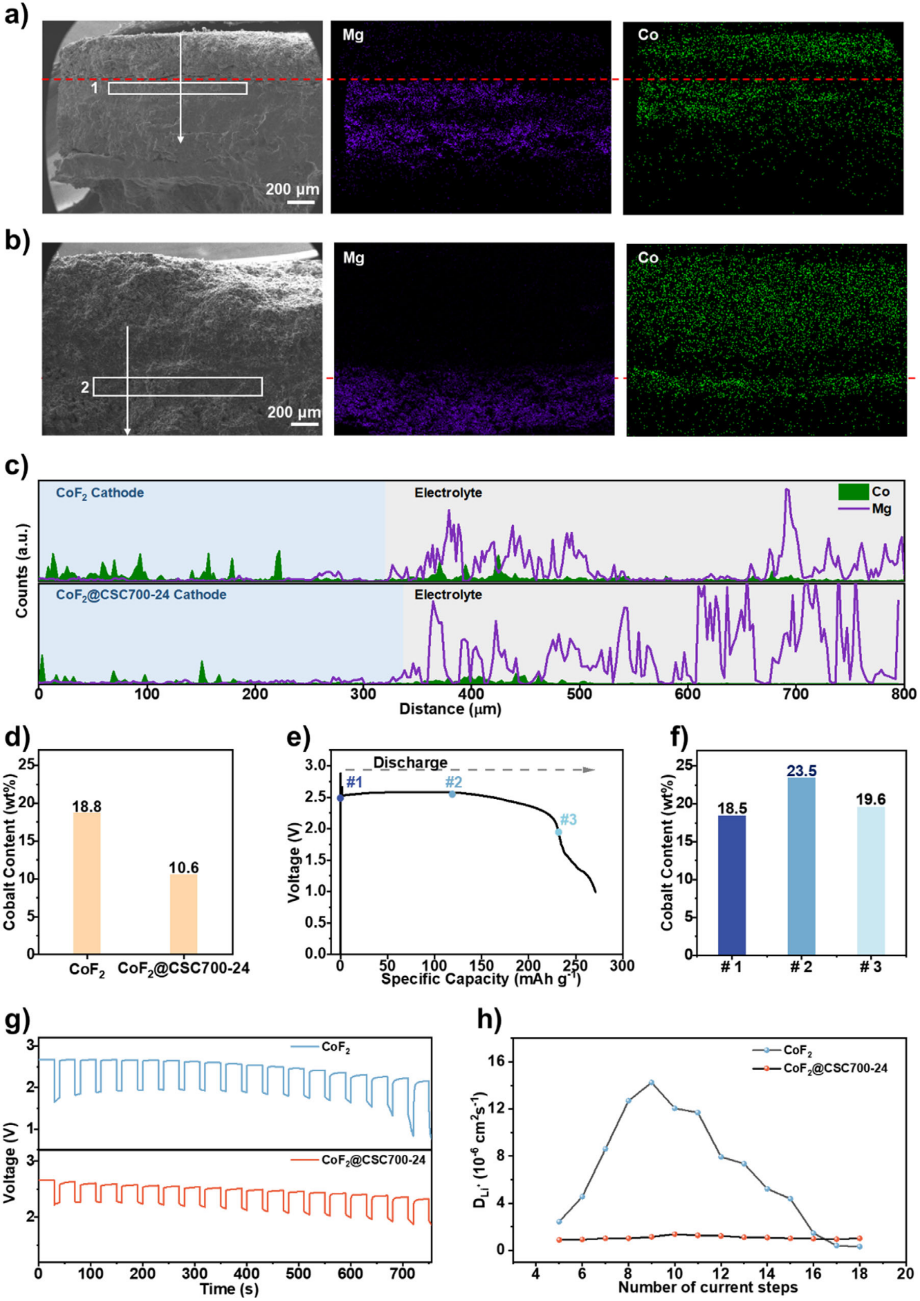
Post‐mortem analysis of dissolved species shuttling within the molten salt. (a,b) FESEM and elemental mapping images of thermal battery cross sections using bared CoF_2_ (a) and CoF_2_@CSC700‐24 (b) as cathode. (c) EDS line scan profiles at marked positions of thermal battery cross sections using bared CoF_2_ and CoF_2_@CSC700‐24 as cathodes. (d) Cobalt contents for the marked zones of thermal battery cross sections using bared CoF_2_ and CoF_2_@CSC700‐24 as cathodes. (e,f) Cobalt contents (f) for electrolyte enrichment zones at different discharging stages (e) of thermal batteries using bare CoF_2_ as cathode. (g) GITT curves of single‐cell thermal batteries with bare CoF_2_ and CoF_2_@CSC700‐24 cathodes, respectively. (h) Li^+^ ion diffusion coefficient at different current steps.

To understand CoF_2_ dissolution during high‐temperature operation and the role of CSC, post‐mortem analysis was conducted on fully discharged and cooled cells. Dissolved species shuttle within the molten salt and precipitate upon electrolyte solidification during cooling. The spatial distribution of elements across the cell cross‐section was analyzed using FESEM and EDS elemental mapping (Figure 5a,b). The boundary between the cathode and electrolyte was identified by the distribution of Magnesium (Mg) from the MgO binder, which remains solid during the whole discharge process. For the cell with a bare CoF_2_ cathode, cobalt was clearly observed shuttling into the electrolyte zone, forming an enrichment layer approximately 300 µm thick, as characterized by EDS line scans (Figure 5c). EDS quantitative analysis within a selected zone (Figure 5a) indicated a cobalt content of 18.8 wt.% in this enrichment zone. In sharp contrast, for the cell using CoF_2_@CSC700‐24 as the cathode, this shuttling was significantly suppressed, resulting in a thinner enrichment zone (∼150 µm, Figure 5b,c) with a lower cobalt content of 10.6 wt.% (Figure 5d). We further employed X‐ray photoelectron spectroscopy (XPS) to measure the carbon/cobalt (C/Co) ratio of cathodes after complete discharge, a technique that probes elemental compositions within a depth of approximately 10 nm. Following full discharge, the CoF_2_@CSC700‐24 cathode exhibited a C/Co ratio of 207.5:1—significantly higher than the 51.8:1 ratio of the bare CoF_2_ cathode (Figure S16). This result indicates that cobalt is effectively confined within the CSC interface during the discharge process.

To investigate the dissolution timeline, cells with bare CoF_2_ cathodes were sampled at different stages: immediately after incubating at 500°C (point #1), mid‐discharge plateau (point #2), and end of the discharge plateau (point #3) (Figure 5e). Significant cobalt shuttling was observed at all three stages, with cobalt content in the electrolyte enrichment zone ranging between 18.5–23.5 wt.% (Figure 5f; Figure S17), indicating that dissolution and shuttling occur rapidly upon electrolyte melting, even before discharging begins. This premature displacement of active material significantly reduces the achievable specific capacity and energy.

To further investigate the impact of the CoCl_4_^2−^ shuttling effect on electrochemical performance, we conducted Galvanostatic Intermittent Titration Technique (GITT) measurements on single‐cell thermal batteries equipped with bare CoF_2_ and CoF_2_@CSC700‐24 cathodes, respectively. As shown in Figure 5g, the CoF_2_@CSC700‐24 cathode exhibited a significantly lower cell voltage drop following current loading, indicating diminished ohmic and charge transfer resistances. Notably, the CoF_2_@CSC700‐24 cathode demonstrated an ultra‐stable Li^+^ ion diffusion coefficient throughout the discharge process, whereas the bare CoF_2_ cathode displayed a drastically fluctuating Li^+^ ion diffusion coefficient—a distinction we attribute to the effective suppression of the shuttling effect and the establishment of stable lithium‐ion transmission channels enabled by the highly porous CSC interface. To investigate the structural stability of the pores, we subjected CSC700 to treatment with NF_3_ at 280°C for 2 h, followed by soaking in an argon atmosphere at 500°C for 0.5 h to simulate the subsequent multi‐step preparation process and the 500°C operating temperature of the battery. Post‐treatment characterization revealed that the pore size of the treated CSC700 remained virtually unaltered, with the distribution still centered at 0.5 nm as determined by CO_2_ physisorption at 273 K (Figure S18).

**FIGURE 6:**
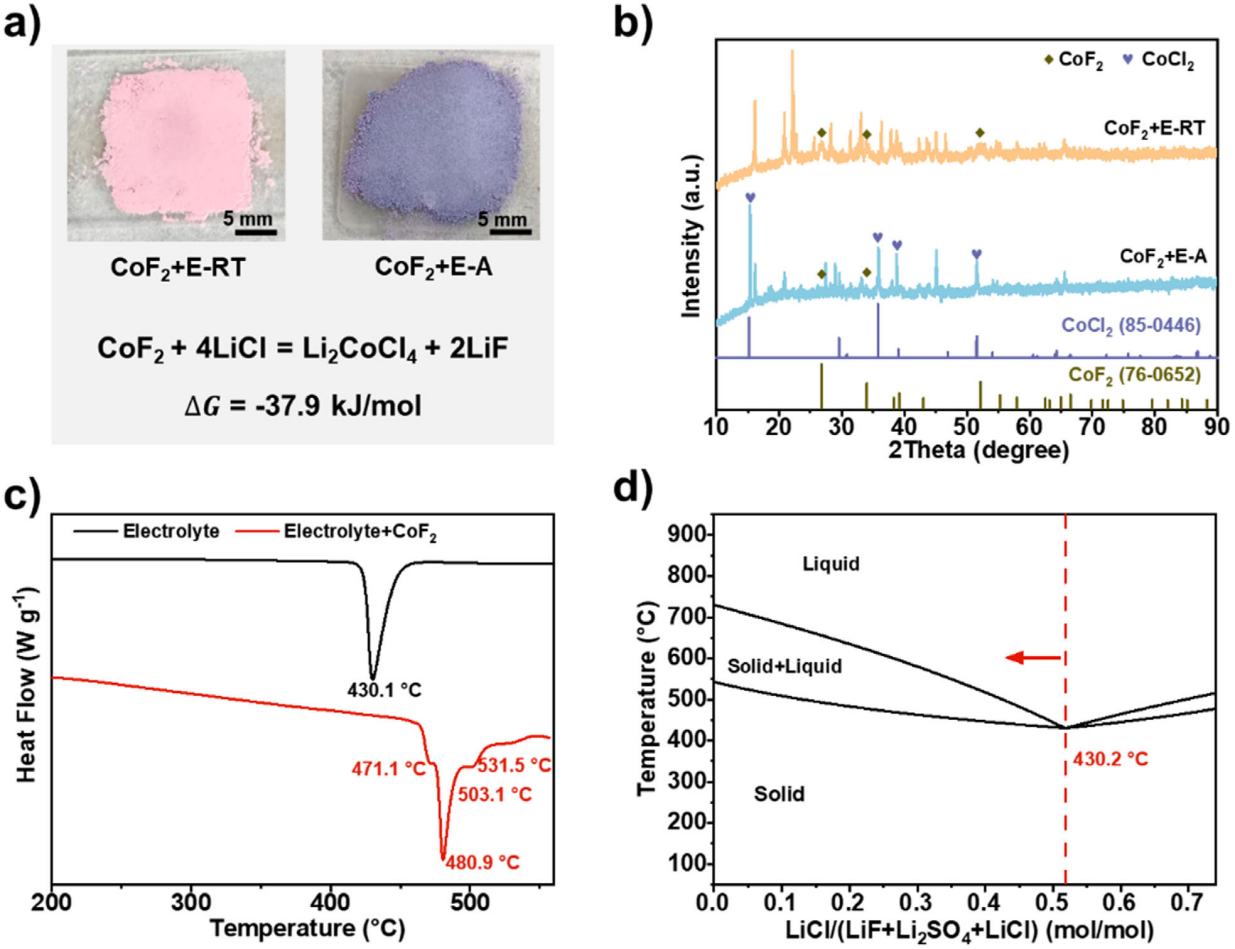
Dissolution mechanism of CoF_2_ in the molten salt electrolyte. (a) photos of CoF_2_+E‐RT and CoF_2_+E‐A and reaction equation with calculated Gibbs free energy of the annealing process. (b) XRD patterns of CoF_2_+E‐RT and CoF_2_+E‐A. (c) DSC curves of pure electrolyte and mixture of electrolyte with CoF_2_ at 3:7 weight ratio. (d) Calculated phase diagram of electrolyte using FactSage.

Recognizing that dissolved metal fluoride species are key, we investigated the dissolution mechanism of CoF_2_ in the molten salt electrolyte. A mixture of CoF_2_ and electrolyte was prepared by grinding at room temperature (CoF_2_+E‐RT) and then annealed at 500°C for 20 min under argon (CoF_2_+E‐A). The color changed from pink to violet after annealing (Figure 6a), characteristic of CoCl_2_. Thermodynamic calculation using the FactSage software predicted the conversion of CoF_2_ with LiCl to form CoCl_4_^2^^−^ at 500°C to be favorable, with a Gibbs free energy of −37.9 kJ mol^−1^ (Figure 6a), suggesting anion exchange due to the high ionicity of the metal‐fluorine bond. This conversion was further evidenced by XRD phase analysis (Figure 6b); after annealing, characteristic peaks of CoF_2_ diminished considerably, while new peaks corresponding to CoCl_2_ appeared. This conversion can further be proved by the XRD pattern of both bared CoF_2_ and CoF_2_@CSC700‐24 cathodes after totally discharged, majorly showing metal Co and LiF (Figure S19). Thus, the primary dissolved species is identified as CoCl_4_^2^^−^ [26], with an estimated diameter of ∼0.8 nm [25], which can be effectively confined by the 0.54 nm pores of the CSC.

This conversion reaction not only affects the cathode but also alters the electrolyte composition by consuming Cl^−^ and releasing F^−^. This shift can change the thermal properties of the electrolyte, a critical parameter for thermal batteries. The ternary LiCl‐LiF‐Li_2_SO_4_ electrolyte used has a single melting point, confirmed experimentally by DSC (melting peak at 430.1°C) and simulatively by FactSage (eutectic point at 430.2°C). However, after mixing with CoF_2_ (3:7 weight ratio), the melting behavior changed significantly, showing a broad melting range with four endothermic peaks centered at 471.1°C, 480.9°C, 503.1°C, and 531.5°C (Figure 6c). This is attributed to the deviation from the eutectic composition, creating a solid‐liquid coexistence region as illustrated in the phase diagram (Figure 6d). Understanding this dissolution mechanism and its impact on both cathode and electrolyte provides new insight into the performance limitations of metal fluoride cathodes in thermal batteries.

## 3 Conclusion

In summary, we developed a selective confinement strategy using a sub‐nanoporous interface to address the dissolution issue of metal fluoride cathodes in thermal batteries employing molten salt electrolytes. The interface was constructed by in situ growth and carbonization of a COF layer on CoF_2_ particles, resulting in a plum pudding@shell structure for the CoF_2_/CSC composites. Detailed characterization confirmed the chemical composition, porous structure, and morphology of the interface. The corresponding thermal battery cells exhibited excellent electrochemical performances, achieving record‐high specific capacity and specific energy among reported high‐voltage thermal battery cathodes. Mechanistic studies demonstrated the effectiveness of the sub‐nanoporous interface in suppressing the shuttling of dissolved metal fluoride species, identified as CoCl_4_^2−^ through experimental and computational studies. This work provides valuable insights into the dissolution mechanism of metal fluorides in molten salts and offers an effective design strategy for next‐generation metal fluoride cathodes, applicable not only to thermal batteries but also to other Li^+^‐conducting battery systems.

## Conflicts of Interest

The authors declare no conflicts of interest.

## Supporting information




**Supporting file**: advs73541‐sup‐0001‐SuppMat.docx.

## Data Availability

The data that support the findings of this study are available from the corresponding author upon reasonable request.
