# Supplementary material for: Selective Confinement by a COF‐Derived Sub‐Nanoporous Interface for High‐Performance CoF2 Thermal Battery Cathodes
Source: Adv Sci (Weinh). 2026 Jan 4;13(14):e21241. doi: 10.1002/advs.202521241 (PMC12970199; doi:10.1002/advs.202521241)
Supplement: Supplementary file 1 — Supporting file: advs73541‐sup‐0001‐SuppMat.docx. [file ADVS-13-e21241-s001.docx]

**Selective Confinement by a COF-Derived Sub-Nanoporous Interface for High-Performance CoF_2_ Thermal Battery Cathodes**

Mengfan Xu,^a,^^b^ Jun Zhang,^b^ Lili Zhao,^b^ Ying Chu,^b^ Furui Luo,^a,b^ Xinping Cao,^a,b^ Shengnan Guo,^b^ Xueying Wang,^b^ Yongping Zhu^b^* and Song Wang^b^*

^a^University of Chinese Academy of Sciences, Beijing 100039, People’s Republic of China

^b^State Key Laboratory of Mesoscience and Engineering, Institute of Process Engineering, Chinese Academy of Sciences, Beijing 100190, People’s Republic of China

*Corresponding authors: ypzhu@ipe.ac.cn; wangsong@ipe.ac.cn

**Section S1. Materials and Instrumentation**

**Materials**

1,3,5-tris(4-aminophenyl) benzene (TAPB, 98%) and 1,3,5-benzenetricarbaldehyde (BTCA,98%) were purchased from ATK Chemical Company Limited. benzaldehyde (98%), aniline (99.5%), scandium (III) trifluoromethanesulfonate (Sc(OTf)_3_, 99%), dioxane (99.5%) and mesitylene (99.5%) were purchased from Sigama-Aldrich. Cobalt power (98%), CoF2 power (98%) and H_2_SiF_6_ acid aqueous solution (30%) were purchased from Aladdin Biochemical Technology. 10% NF_3_/90%Ar was purchased from New Red Specialty Gases Co., Ltd. All chemicals and solvents were directly used without any further purification.

**Instrumentation**

**Field emission scanning electron microscopy (FESEM).** FESEM images were obtained from a JMS-7800 field emission scanning electron microscope. The accelerating voltage was set as 15 kV. All the samples were coated with Pt for 180 s.

**Transmission electron microscope (TEM).** TEM and HRTEM images were obtained by a JEOL-JEM-ARM200F transmission electron microscope. The acclerating voltage was set as 200 kV. Samples was attached to a coppor grid covering ultrathin carbon layer by dipping the grid onto the dry powder and followed by nitrogen purging to remove excess powder.

**X-ray diffraction (XRD).** XRD spectrum was collected with a Rigaku SmartLab 9 kW X-pert Powder diffractometer (Cu anode). Scan step was set as 0.0100° and scan speed 10 °/min.

**N_2_ absorption-desorption analysis.** The absorption-desorption isotherms was collected by a Micromeritics 3Flex analyzer at 77 K. The sample was outgassed at 120 °C for 12 hours before measurement. The Brunauer-Emmett-Teller (BET) surface area and pore width were calculated on MicroActive software.

**CO_2_ absorption-desorption analysis.** The absorption-desorption isotherms was collected by a Micromeritics 3Flex analyzer at 273 K. The sample was outgassed at 120 °C for 12 hours before measurement. The Brunauer-Emmett-Teller (BET) surface area and pore width were calculated on ASiQwin software.

**Simultaneous thermal analyzer.** The DSC and TGA curves were obtained by a TA Instruments SDT Q600. Samples were kept at 100 °C for 30 min and then heated to 900 °C target temperature at a rate of 10.0 °C /min. The atmosphere was N_2_ or air accordingly.

**Flourier transformed infrared (FT-IR)** **spectroscopy.** FT-IR spectra were collected on a Tensor II spectrometer from Bruker.

**Laser Raman spectroscopy.** Raman spectra were collected on a in Via spectrometer from Renishaw. Excitation Wavelength was 523 nm. The Raman range was 100~400 cm^-1^.

**X-ray photoelectron spectroscopy. XPS** spectra were collected on a in ESCALAB 250Xi from Thermo Fisher Scientific.

**Section S2. Experimental procedures**

**Synthesis of TAPB-BTCA COF and carbonization with different temperatures**

In a typical experiment with 5.00 mM of monomers in dioxane/mesitylene (1:1, v/v), 17.6 mg of TAPB (0.05 mmol, 1equiv) was dissolved in 5 mL of dioxane/mesitylene (1:1, v/v), followed by adding 60.7 μL benzaldehyde (0.6 mmol, 12 equiv) to produce solution A. BTCA (8.1 mg, 0.05 mmol) was dissolved in 5 mL of dioxane/mesitylene (1:1, v/v), followed by adding 54.4 μL of aniline (0.6 mmol, 12 equiv.) to produce solution B. Section A and B were the mixed, and Sc(OTf)_3_ (0.73 mg, 1.5 μmol) in 0.1 mL of dioxane/mesitylene (1:1, v/v) were added, and the system was allowed to react for 3 days. A yellow power was produced, which was isolated through filtration and washing with dioxane/mesitylene (1:1, v/v) to remove the residual monomers. After drying at ambient condition for 1 days naturally and under vacuum at 40 °C for another day, final products were obtained.

A certain amount of TAPB-BTCA COF is tidily placed in a ceramic boat, heated to 500 °C, 600 °C, 700 °C and 800 °C with a ramp rate of 3 °C min^-1^, and maintained for 3h in pure Argon in a tube furnace, respectively. After that, the furnace is cooled to room temperature naturally. The color of the final samples is black except for the one at 500 °C, which is brown.

**Synthesis of CoF_2_@CSC composites**

In a typical synthesis process, Cobalt powers (1.2 g) were reacted in 10 g (30 wt%) H_2_SiF_6_ acid aqueous solution for 24 h at room temperature. After the reaction was completed, the solution was centrifuged to remove the excess solids and dried at room temperature 24 h to obtain power. Then the power was annealed at 260 °C for 4 h under Argon flow.

A portion of the CoF_2_ power was then added into different proportions of A and B solution and Sc(OTf)_3_ in dioxane/mesitylene (1:1, v/v) were added, with stirring continuously, and the system was allowed to react for 3 days.A yellow power was produced, which was isolated through filtration and washing with dioxane/mesitylene (1:1, v/v) to remove the residual monomers. After drying at ambient condition for 1 days naturally and under vacuum at 40 °C for another day, CoF_2_@COF samples were obtained.

In a typical experimental, a certain amount of CoF_2_@COF composite is placed in a ceramic boat and heated to certain temperatures with a ramp rate of 3 °C min^-1^, and maintained for 3 h under Argon flow in a tube furnace, respectively. After that, the furnace is cooled to room temperature naturally.

A portion of carbonized different carbonization temperatures of CoF_2_@COF is placed in a ceramic boat and heated to 280 °C with a ramp rate of 5 °C min^-1^ and maintained for 2 h in NF_3_/Ar (10% NF_3_ in Argon) in a tube furnace. Then, the furnace is cooled to room temperature, and the samples CoF_2_@CSC composites were finally obtained and stored in a glovebox. The carbon contents in CoF_2_@CSC were 21%, 22%, 22%, and 23% obtained via the elemental analysis.

The same procedure was used for synthesizing carbonized different proportions of CoF_2_@COF. A certain amount of CoF_2_@COF composite is placed in a ceramic boat and heated to 700 °C with a ramp rate of 3 °C min^-1^, and maintained for 3 h under Argon flow in a tube furnace. After that, the furnace is cooled to room temperature naturally.

A portion of carbonized different coating ratios of CoF_2_@COF is placed in a ceramic boat and heated to 280 °C with a ramp rate of 5 °C min^-1^ and maintained for 2 h in NF_3_/Ar (10 % NF_3_ in Argon) in a tube furnace. Then, the furnace is cooled to room temperature, and the samples CoF_2_@CSC700-11, CoF2@CSC700-15, CoF_2_@CSC700-19, CoF_2_@CSC700-24 and CoF_2_@CSC700-27 composites were finally obtained and stored in a glovebox. The carbon contents in CoF_2_@CSC were obtained via the elemental analysis. The commercial CoF_2_ particles purchased from Aladdin Biochemical Technology were used for performance comparison.

**Preparation of single cells and discharge tests**

A single cell consists of Li-B alloy (Li 60 wt.%) sheets as anode, the mixture of 50% MgO binder and 50% LiCl-LiF-Li_2_SO_4_ eutectic salt as the separator, the mixture of 70 wt.% CoF_2_ and 30% wt.% LiCl-LiF-Li_2_SO_4_ separator as cathode. There are 0.105 g of CoF_2_@CSC, 0.045 g of LiCl-LiF-Li_2_SO_4_ and 0.01 Super P existing in every cathode platelet (0.16 g). The quality of the Li-B alloy sheet and separator with every single cell is 0.06 g and 0.16 g, respectively. The cathode and the separator are pressed into Փ12 mm pellets under 30 MPa. Areal mass loading of CoF_2_ is 92.84 mg/cm^2^. Total mass of battery is 0.38 g. Afterwards, the single cells are assembled in glove-box filled with argon, in which the moisture and oxygen concentration is controlled under 5 ppm. The discharge performance of cells is examined temperature of 500 °C with various current densities of 100 mA cm^-2^, 300 mA cm^-2^ and 500 mA cm^-2^, which were conducted on battery testers (LandCT3002K). And the cells are contained at the test temperature for around 10 s before the load is applied to ensure the cells are heated to the given temperature. The test system is self-developed according to literature. Based on a great number of works, this system is evidenced reliable and reproducible, which can truthfully represent the discharge performance of the cathode materials.

The lithium-ion migration was calculated using the equation below:

$$D=\frac{4}{\pi\tau}{(\frac{m_{B}V_{M}}{SM_{B}})}^{2}{(\frac{\Delta E_{s}}{\Delta E_{\tau}})}^{2}$$

Here, *m_B_, V_M_* and *M_B_* denote the mass, molar volume, and molar mass of the cathode's active material, respectively. The parameter *S* represents the contact area between the cathode material and the electrolyte. $\Delta E_{s}$ is the voltage difference between the endpoints of two adjacent relaxation phases, and $\Delta E_{\tau}$ refers to the total voltage change during the constant-current discharge phase.

**
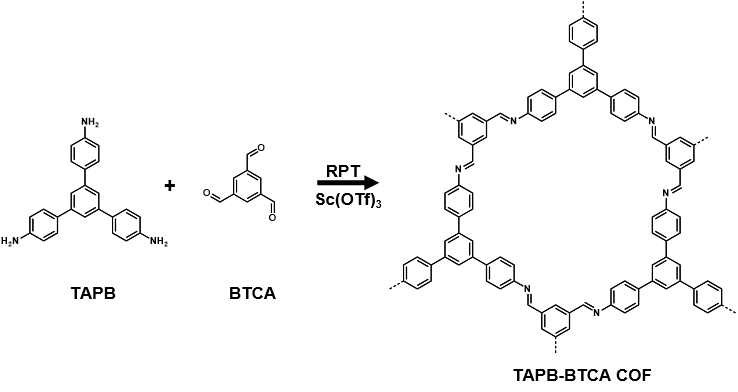
**

**Figure S1.** Chemical reaction of TAPB-BTCA COF synthesized from TAPB and BTCA monomers via RPT method

**Figure S2.** FTIR spectrum of TAPB, BTCA and TAPB-BTCA COF


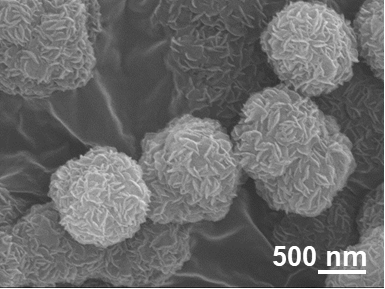


**Figure S3.** FESEM image of TAPB-BTCA COF

**Figure S4.** Thermal analysis curves of TAPB-BTCA COF

**Figure S5.** Raman spectrum of TAPB-BTCA COF, the fluorescence is so strong that the intensity was over range after Raman shift of 1000 cm^-1^


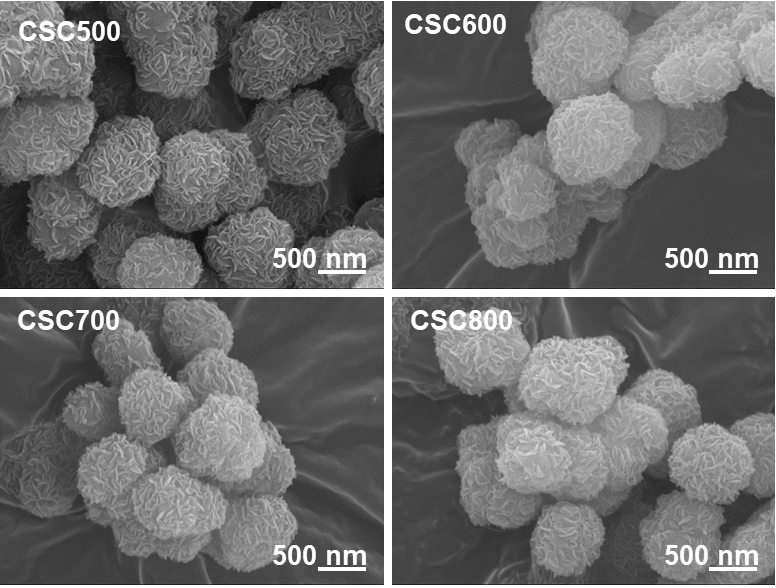


**Figure S6.** FESEM images of CSC500, CSC600, CSC700 and CSC800

**Figure S7.** XRD pattern of CoSiF_6_ 6H_2_O

**Figure S8.** Thermal analysis curves of CoSiF_6_ 6H_2_O

**
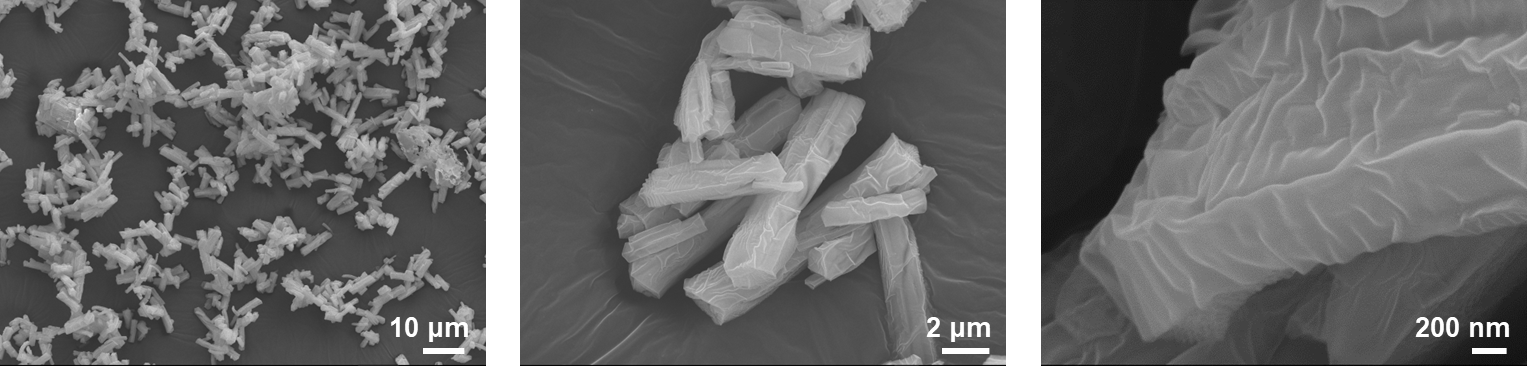
**

**Figure S9.** FESEM images of CoSiF_6_ 6H_2_O


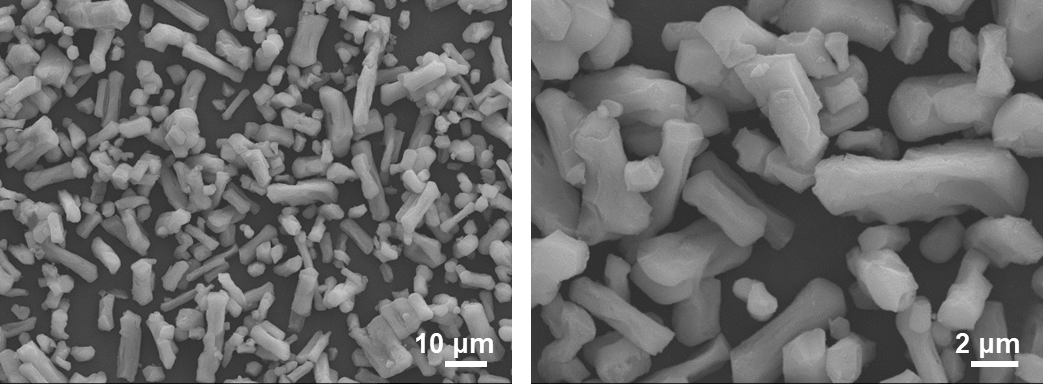


**Figure S10.** FESEM images of CoF_2_


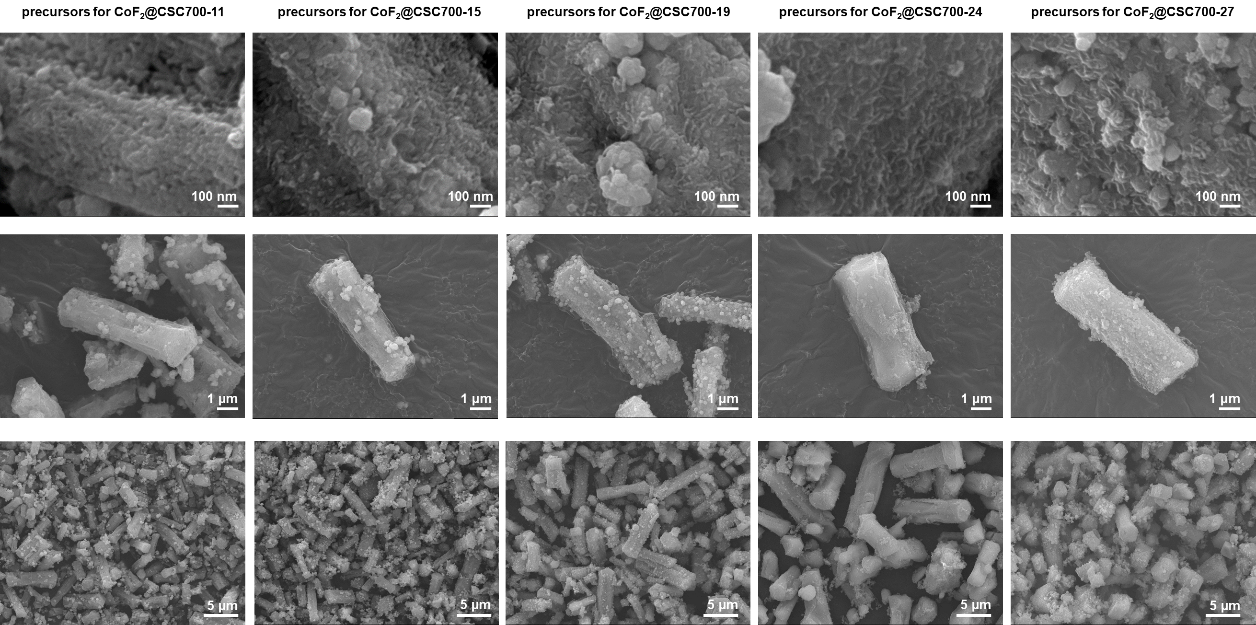


**Figure S11.** FESEM images of CoF_2_@TAPB-BTCA COF precursors for CoF_2_@CSC700s

**Figure S12.** XRD patterns of CoF_2_@CSC700-11, CoF_2_@CSC700-15, CoF_2_@CSC700-19, CoF_2_@CSC700-24, and CoF_2_@CSC700-27 before subsequent fluorination with NF_3_. Characteristic peaks belonging to Metal Co can be observed, which can be attributed to the reduction by carbon during the carbonization


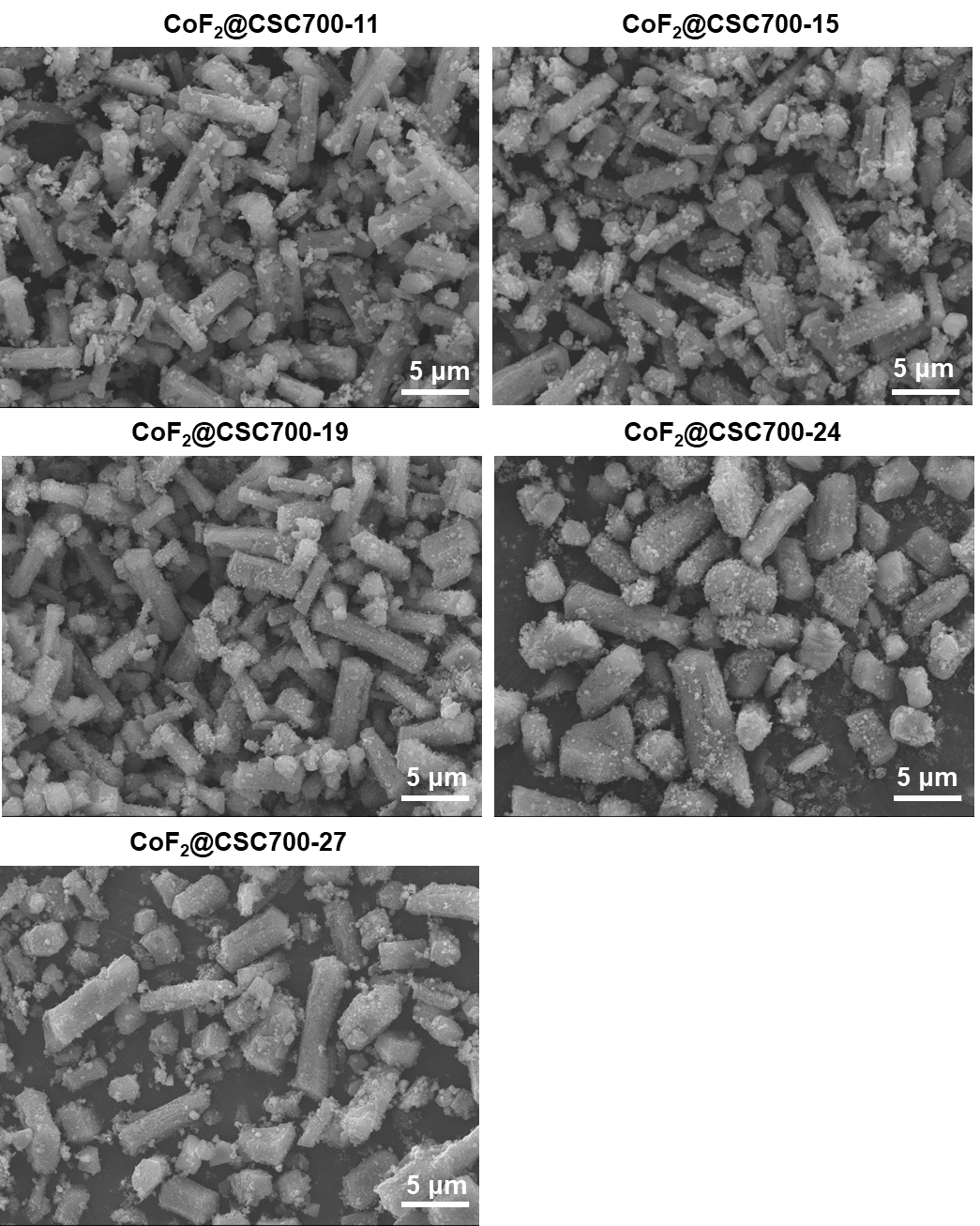


**Figure S13.** FESEM of CoF_2_@CSC700-11, CoF_2_@CSC700-15, CoF_2_@CSC700-19, CoF_2_@CSC700-24 and CoF_2_@CSC700-27


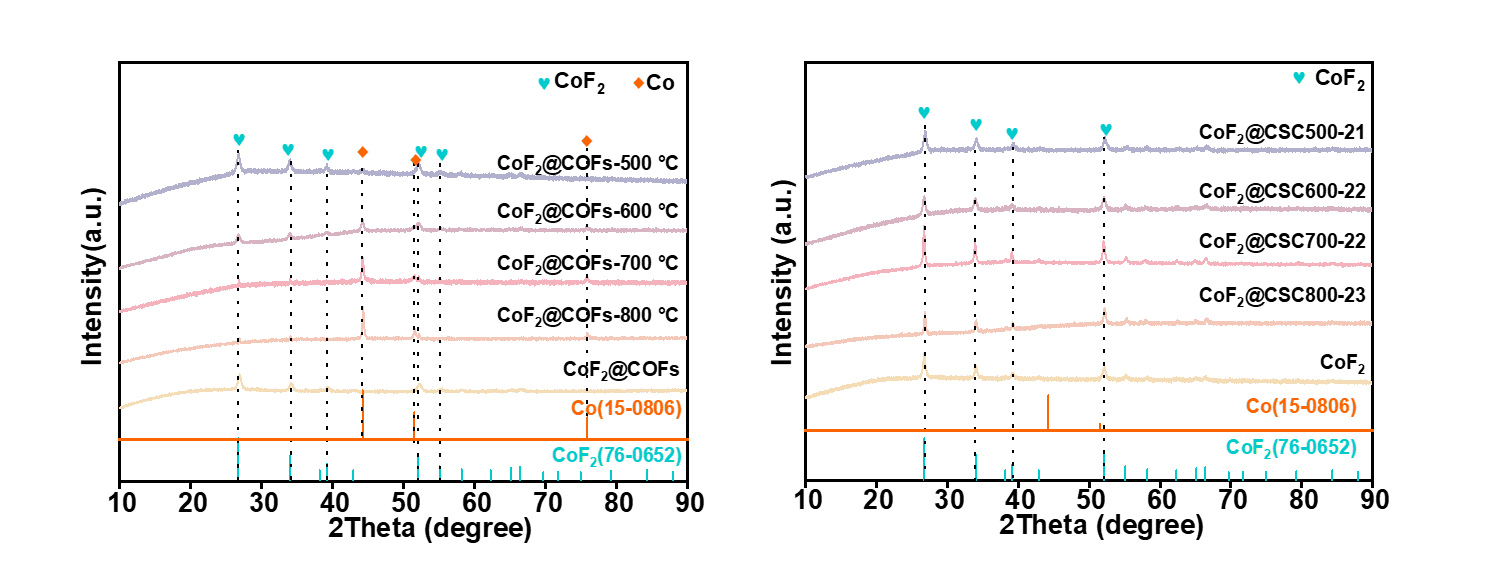


**Figure S14.** XRD patterns of CoF_2_@CSC500-21, CoF_2_@CSC600-22, CoF_2_@CSC700-22, CoF_2_@CSC800-23 before (left) and after (right) subsequent fluorination with NF_3_

**
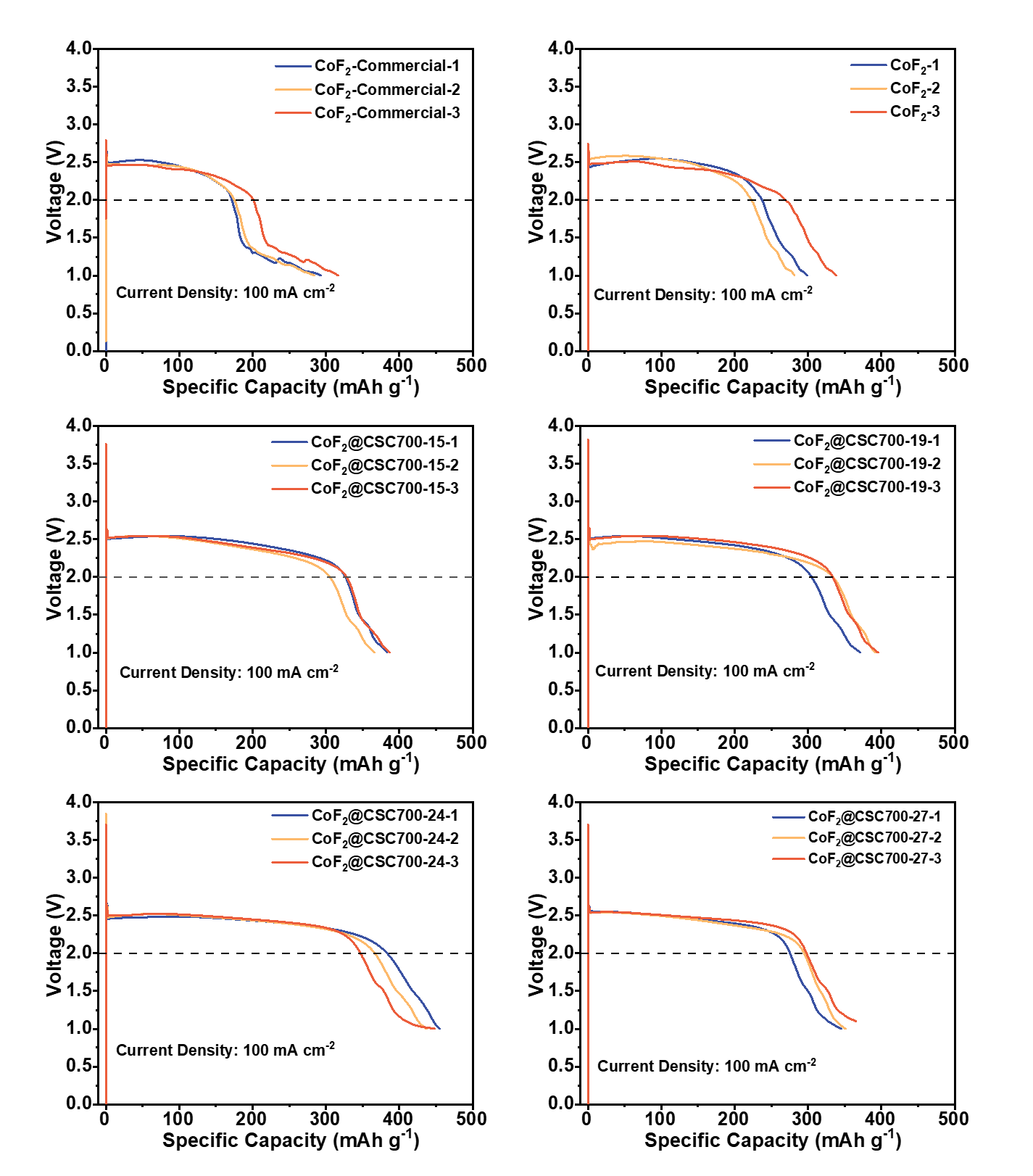
**

**Figure S15.** The discharge behavior of single cell thermal batteries with CoF_2_@CSC700-11, CoF_2_@CSC700-15, CoF_2_@CSC700-19, CoF_2_@CSC700-24 and CoF_2_@CSC700-27 cathodes at a constant current density of 100 mA cm^-2^ and a temperature of 500 °C


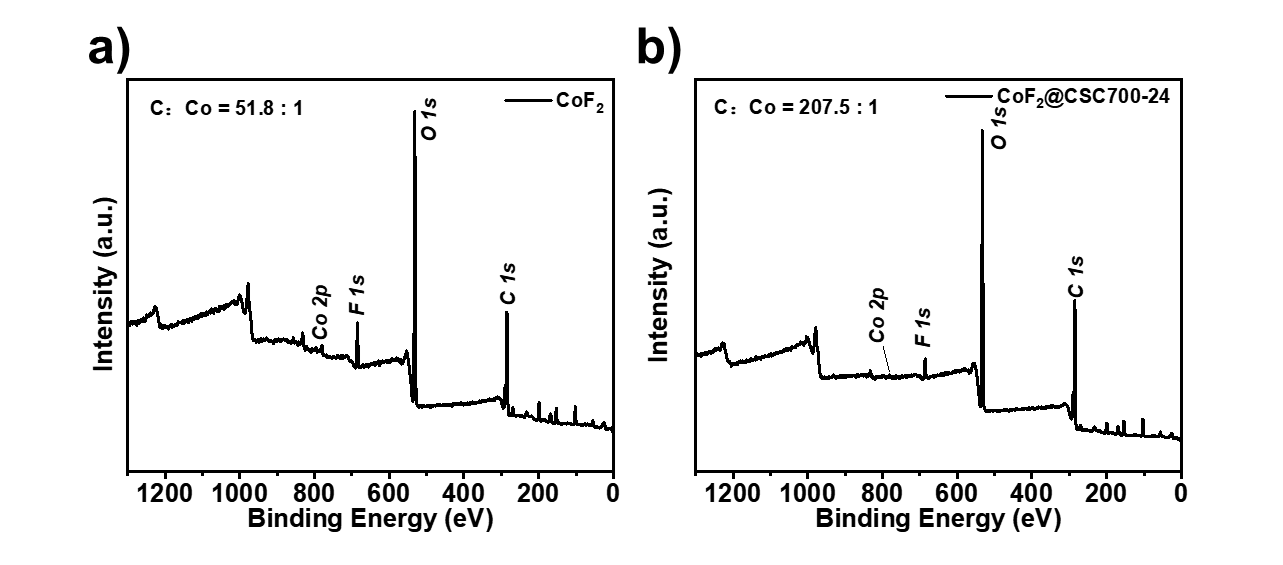


**Figure S16.** XPS spectrums of bare CoF_2_ (a) and CoF_2_@CSC700-24 (b) cathodes after discharge

**
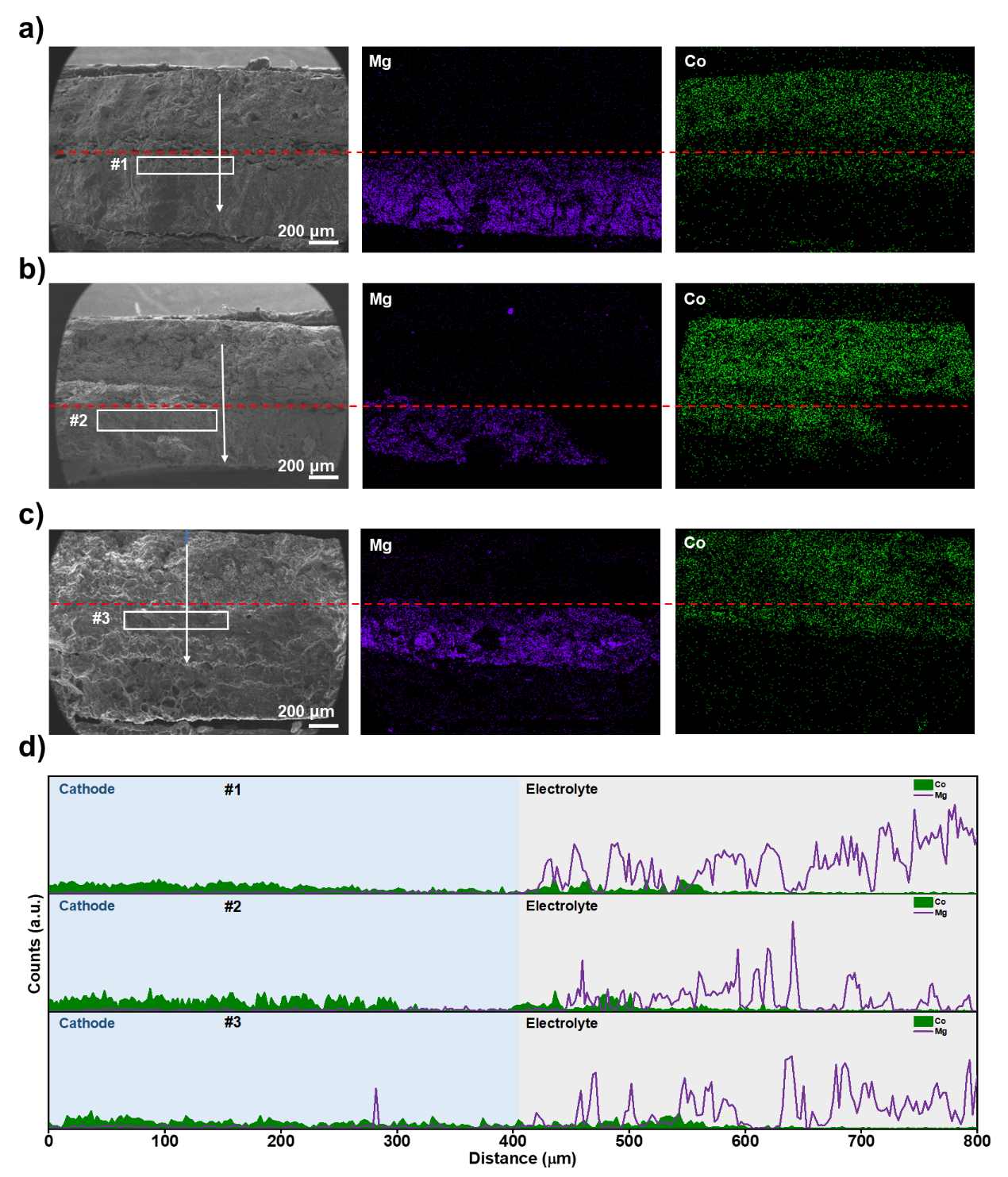
**

**Figure S17.** Post-mortem analysis of dissolved species migration within the molten salt at different discharging stages. a-c) FESEM and elemental mapping images of thermal battery cross sections at discharging stage #1 (a), #2 (b) and #3 (c). d) EDS line scan profiles at marked positions of thermal battery cross sections at different discharging stages


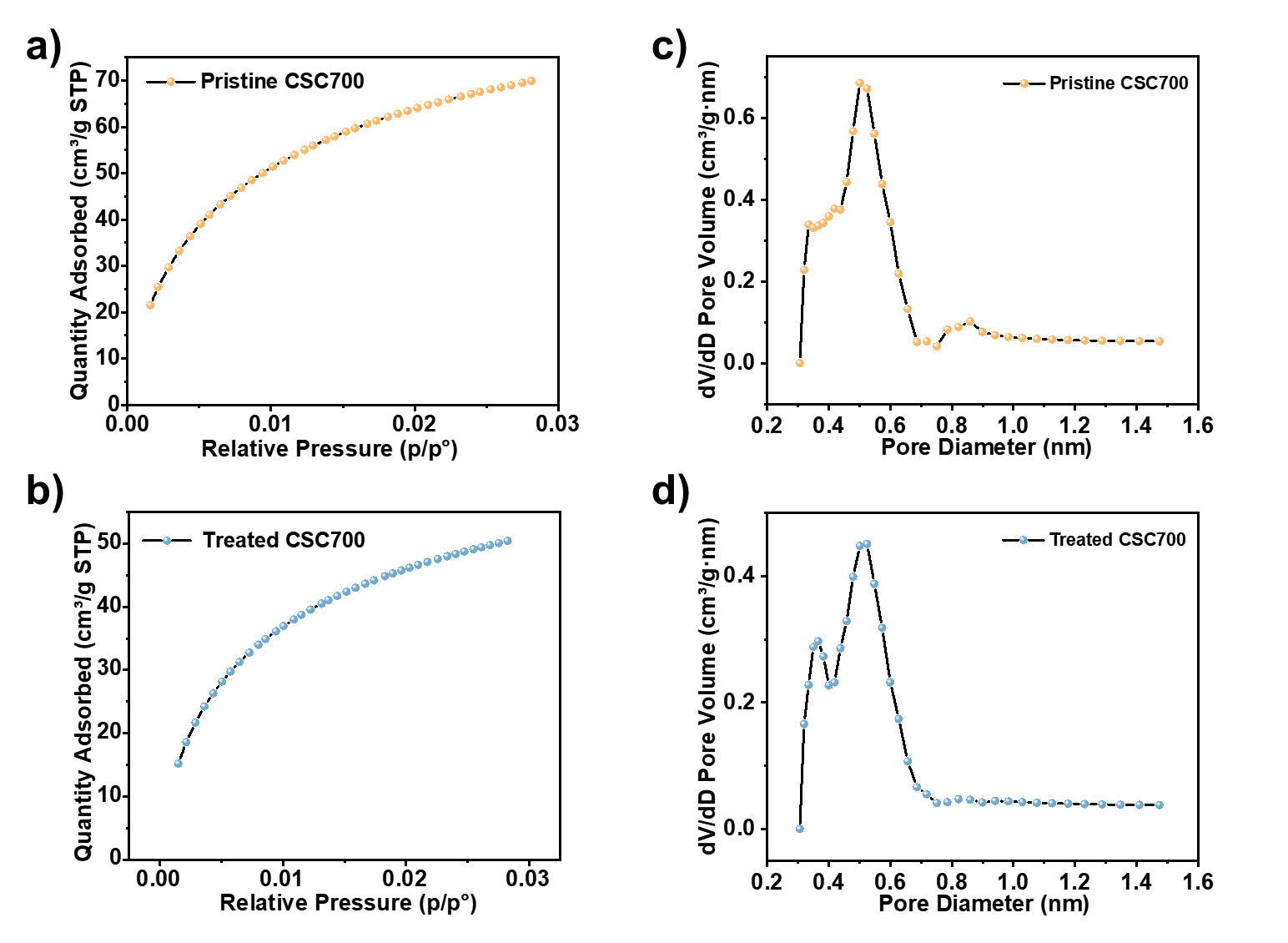


**Figure S18.** CO_2_ physisorption curves at 273 K for pristine (a) and treated CSC700 (b). Pore size distributions of pristine (c) and treated CSC700 (d) using the DFT method based on CO_2_ physisorption at 273 K

**Figure S19.** XRD patterns of totally discharged bare CoF_2_ and CoF_2_@CSC700-24 cathodes
